# Application of Pain Management After Lumbar Spine Surgery: A Bibliometric Analysis of Randomized Controlled Trials

**DOI:** 10.1155/prm/3601838

**Published:** 2026-09-08

**Authors:** Duoduo Li, Yena Gan, Xia Li, Sheng Han, He Zhu, Caoyuan Yang, Yujia Zhang, Ranya Huang, Qifei Li, Anagul Tudi, Bofei Meng, Changxin Liu, Xing Yu

**Affiliations:** ^1^ Dongzhimen Hospital, Beijing University of Chinese Medicine, Beijing, China, bucm.edu.cn; ^2^ Surgery and Cancer, Imperial College London, London, UK, imperial.ac.uk; ^3^ Department of Academic Research, International Research Center for Medicinal Administration, Peking University, Beijing, China, pku.edu.cn; ^4^ Centre for Evidence Based Chinese Medicine, Beijing University of Chinese Medicine, Beijing, China, bucm.edu.cn; ^5^ Changxin Pain Rehabilitation Physician Group, Shenzhen, China

**Keywords:** Asian, bibliometric analysis, lumbar, numeric rating scale, pain, postoperative, randomized controlled trials, spine, surgery, Visual Analog Scale

## Abstract

**Study Design:**

Bibliometric analysis.

**Objective:**

To optimize pain management and identify future research trends through bibliometric analysis.

**Summary of Background Data:**

Postoperative lumbar spine pain is multifactorial and often persists despite successful surgery, reducing patients’ quality of life, increasing healthcare use, and increasing the risks of opioid dependence and psychological distress.

**Methods:**

Data from eligible studies were extracted through a comprehensive search and various analyses, including descriptive bibliometrics, citations, keywords, and thematic analyses. Group comparisons were made between Asian and non‐Asian groups to assess differences and trends between the two regions.

**Results:**

Between 1984 and 2025, 197 studies were published, with an average annual increase of 1.70%. At least 10 studies were contributed by the USA, Turkey, Iran, China, and Korea. Studies from the USA had the highest annual citations, whereas those from Spain had the highest citations per article. The focus of most studies (96.45%) was on pharmacological interventions, including anesthetics, opioids, nonsteroidal anti‐inflammatory drugs, antipyretic–analgesic drugs, corticosteroids, serotonin–norepinephrine reuptake inhibitors, nonopioid centrally acting analgesics, and nondepolarizing neuromuscular blockers. Among these, intravenous administration was used in 48.22% of the studies, and 58.38% employed a placebo as the control. Additionally, 77.16% of the studies used the Visual Analog Scale to assess pain. Overall, the field has shifted from basic pain management to more personalized and multifaceted perioperative strategies, especially for patients undergoing complex spinal surgeries. Significant differences were observed between the Asian and non‐Asian groups regarding publication year and age.

**Conclusions:**

The number of randomized controlled trials on lumbar postoperative pain has increased, with the USA, Turkey, Iran, and Korea dominating the research. China’s lower citation ranking was lower on citation‐based indicators; limited international visibility is proposed as a hypothesis for this pattern rather than an established explanation. Driven by the opioid crisis, the field has evolved from opioid‐sparing strategies to multimodal, personalized care.

## 1. Introduction

Postoperative pain is a common concern following lumbar disc surgery [1]. Approximately 80% of patients experience significant pain relief within the first 3 days postoperatively; however, about 20% continue to suffer from persistent pain [2]. Importantly, this ongoing discomfort can occur even in patients who have undergone technically successful decompression with no evidence of residual nerve compression on postoperative imaging, indicating that structural abnormalities alone do not fully explain the persistent pain [3]. Instead, postoperative lumbar spine pain may have a multifactorial origin, involving structural factors, such as residual or recurrent disc herniation, ongoing nerve root compression, and spinal instability; surgical factors, including inadequate decompression; and psychosocial influences, including anxiety, depression, and pain catastrophizing, all of which can significantly affect pain perception and its persistence [4–6].

Postoperative pain after lumbar spine surgery can severely reduce quality of life, leading to disability, depression, and impaired daily functioning [7–9]. It is commonly treated using conservative methods (medications, physical therapy, and psychotherapy), surgical interventions, and neuromodulation techniques (spinal cord and dorsal root ganglion stimulation, among others) [10–12]. Surgical reintervention is considered for structural issues, but its success rates decrease with repeated procedures [13]. Treatment effectiveness varies with factors such as surgery type, comorbidities, and pain severity [14]. Current approaches can reduce pain and aid recovery; nonetheless, they often fail to address root causes like nerve damage or psychological factors [6, 15]. Limitations such as adverse effects (AEs), drug tolerance, declining neuromodulation efficacy, and the lack of standardized multimodal protocols contribute to persistent pain in many patients and increased healthcare burden [16–18], leading to greater healthcare utilization, higher costs, and a greater risk of long‐term opioid dependence and psychological distress [14, 19].

Therefore, a comprehensive understanding of the research landscape is necessary. Bibliometric analysis can be used to systematically examine the existing literature, identify major research trends, highlight influential countries and authors, and reveal evidence gaps, particularly regarding multimodal treatment strategies. By analyzing publication patterns and citation data, the aim of the study was to guide future research priorities, inform clinical decision‐making, and promote the development of more effective, evidence‐based, and personalized approaches to pain management.

## 2. Methods

In May 2025, Scopus, Web of Science Core Collection, PubMed, Embase, and the Cochrane Library databases were comprehensively searched, using keywords and synonyms related to “pain management” and “lumbar spine surgery.” Keywords were combined using the Boolean operators (“AND” and “OR”), and database‐specific search syntax was adapted according to the indexing requirements of each database while maintaining equivalent search concepts. The complete search strategies and database‐specific search strings are provided in Additional File 1. The research protocol was registered in the International Prospective Register of Systematic Reviews (PROSPERO, CRD420251078972). After removing duplicate records, two reviewers (D.L. and Y.G.) independently screened the titles and abstracts for eligibility, and full texts were assessed when necessary. Disagreements were resolved through discussion with a third reviewer (X.Y.).

Randomized controlled trials (RCTs) involving patients undergoing lumbar spine surgery were included, and pharmacological or nonpharmacological interventions for postoperative pain management were assessed. Studies were required to report outcomes, such as pain intensity, opioid use, or functional recovery, and to include a comparator such as a placebo, best standard care, or alternative treatment. Only peer‐reviewed, full‐text RCTs published in English were included in the analysis. Nonlumbar surgeries, intraoperative anesthesia without postoperative pain data, non‐RCT designs, reviews, abstracts, and animal studies were excluded.

Data cleaning, performed before bibliometric analysis and data extraction, included checking bibliographic records for consistency, removing noninformative keywords (e.g., “article”), and standardizing synonymous keywords to improve consistency across studies. The cleaned dataset was manually verified to ensure the accuracy and completeness of the extracted information. The article data, including publication year, authors, country/region, title, journal, Journal Citation Reports (JCR) division, impact factor (IF), keywords, references, and citations, were collected using a predesigned Microsoft Excel spreadsheet. The extracted data were independently checked by two reviewers, and any discrepancies were resolved through discussion to ensure data accuracy. Data on participant demographics (sample size, sex, and age), protocols (agent type and relevant details), and outcome indicators were extracted. These outcome indicators included the following: Visual Analog Scale (VAS), Numeric Rating Scale (NRS), Verbal Rating Scale (VRS), Neuropathic Pain Scale (NPS), AEs following pharmacologic intervention, postoperative supplementary analgesics consumption, spinal instability, patient satisfaction, Oswestry Disability Index (ODI), heart rate, blood pressure, respiratory rate, oxygen saturation (SpO2), Roland‐Morris Disability Questionnaire (RMDQ), SF‐McGill Pain Questionnaire, Athens Insomnia Scale, Beck Depression Inventory II, Brief Pain Inventory, Color Analysis Scale, Hospital Anxiety and Depression Scale (HADS), Health‐Related Quality of Life (HRQL), EuroQol‐5 Dimensions (EQ‐5D), Oldenburg Burnout Inventory (OLBI), arterial carbon dioxide partial pressure (PaCO2), pain intensity difference (PID), Pittsburgh Sleep Quality Index, State Anxiety Inventory, and Tampa Scale for Kinesiophobia (TSK).

**FIGURE 1:**
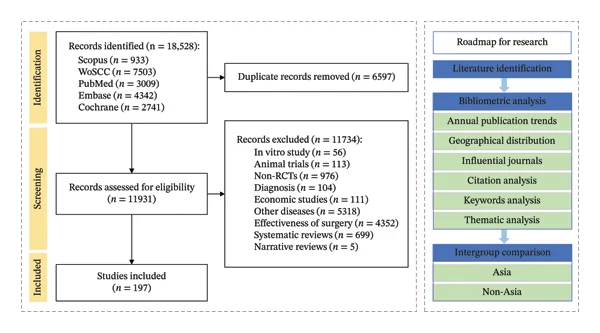
Flowchart of study selection and research methodology.

The annual and global distributions of studies in influential journals, countries, and emerging trends in topics were assessed. Descriptive bibliometrics, citations, keywords, and thematic analyses were conducted. Differences in publication year, sex, age distribution, pain management timing, and interventions between the Asian and non‐Asian groups were assessed. Bibliographic data were mapped using the Bibliometrics and ggplot packages in R software (Ver. 4.3.1). The study selection process and methodology are illustrated in the flowchart (Figure 1).

## 3. Results

**FIGURE 2:**
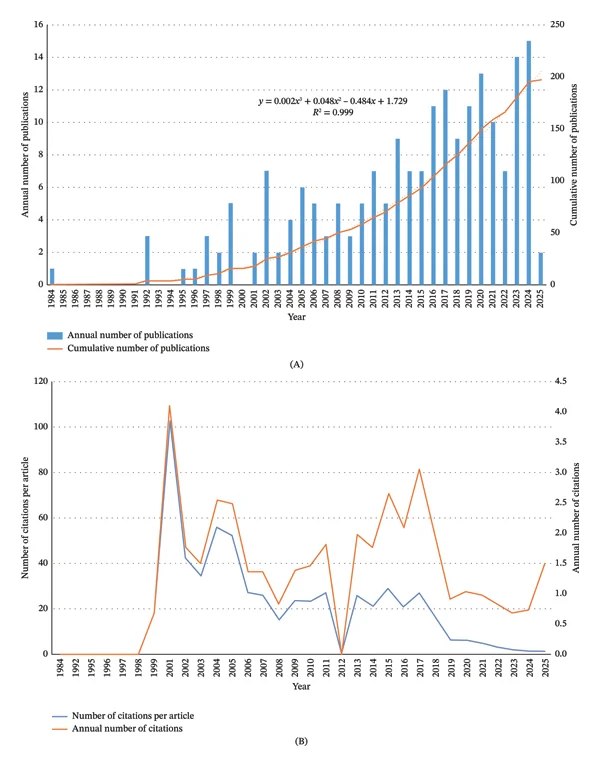
Trends in publication count and citation frequency. (A) Annual trends in publication count. (B) Annual trends in citation frequency.

Overall, 197 studies conducted between 1984 and 2025, published in 101 journals, and authored by 1048 individuals from 36 countries, were identified. The cumulative number of publications exhibited an exponential trend (*y* = 0.002*x* ^4^ + 0.048*x* ^3^ − 0.484*x* ^2^ + 1.729, *R* ^2^ = 0.999), with a 1.70% average annual increase in publications (Figure 2A). Citations per article and average annual citations showed declining trends, especially between 2001 and 2012 (Figure 2B).

**FIGURE 3:**
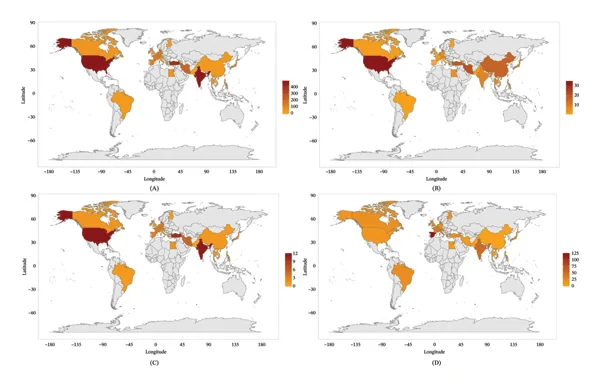
The geographic contribution of countries. (A) Overall publications. (B) Overall citations. (C) Mean citations per year. (D) Mean citations per publication.

The USA, Turkey, Iran, China, and Korea contributed a minimum of 10 articles each. Except for China, articles from these countries ranked highest in citations (Table 1). Studies from the USA had the highest average of 12 citations per year, whereas those from Spain had the highest average of 125 citations per article (Figure 3).

**TABLE 1:** Most productive and influential countries and journals.

Rank	Country productivity (publications)	Country influence (citations)	Journal productivity (publications)	Journal influence (citations)
1	USA (35)	USA (497)	Spine (19)	Journal of Neurosurgical Anesthesiology (450)
2	Iran (21)	India (475)	Journal of Neurosurgical Anesthesiology (13)	Spine (429)
3	Turkey (19)	Turkey (403)	BMC Anesthesiology (7)	Anesthesia and Analgesia (195)
4	China (17)	Korea (374)	European Spine Journal (7)	Canadian Journal of Anaesthesia/Journal Canadien D Anesthesie (184)
5	Korea (14)	Iran (226)	Anesthesia and Analgesia (6)	Anesthesiology (165)
6	India (9)	Denmark (198)	Medicine (5)	Pain (161)
7	Japan (8)	Germany (163)	Spine Deformity (5)	European Spine Journal (148)
8	Thailand (8)	France (153)	ACTA Neurochirurgica (4)	Neurosurgery (139)
9	Germany (7)	Spain (125)	Trials (4)	BMC Anesthesiology (131)
10	Egypt (6)	Japan (95)	ACTA Anaesthesiologica Scandinavica (3)	Clinical Journal of Pain (120)

Most of the authors (93.70%) contributed to a single study. One‐third of the articles were published in the top 10 journals in this field, including *Spine* (Q2, IF = 3.5, 2024), *Journal of Neurosurgical Anesthesiology* (Q2, IF = 2.4, 2024), *BMC Anesthesiology* (Q2, IF = 2.6, 2024), *European Spine Journal* (Q2, IF = 2.7, 2024), *Anesthesia and Analgesia* (Q1, IF = 3.8, 2024), *Medicine* (Q2, IF = 1.4, 2024), *Spine Deformity* (Q3, IF = 1.8, 2024), *ACTA Neurochirurgica* (Q3, IF = 1.9, 2024), Trials (Q3, IF = 2, 2024), and *ACTA Anaesthesiologica Scandinavica* (Q2, IF = 2, 2024) (Table 1).

The focus of the top 10 cited papers was on optimizing multimodal analgesia strategies for postoperative pain management following spinal surgery (Table 2). Preemptive use of gabapentin (300–1200 mg) and pregabalin (300 mg/day) has been frequently reported, demonstrating significantly reduced pain scores and opioid consumption, particularly of fentanyl and morphine, within the first 24 h postsurgery, with minimal adverse events. Furthermore, pregabalin showed slightly superior effects on functional outcomes and quality of life 3 months postoperatively [20, 27, 29]. The use of propacetamol, a prodrug of acetaminophen, showed a 46% reduction in morphine use while maintaining analgesic efficacy and reducing sedation, highlighting its opioid‐sparing effect [21]. Similarly, using low‐dose ketorolac alongside patient‐controlled analgesia (PCA) morphine improved analgesia and reduced morphine requirements without AEs or impaired spinal fusion outcomes [24]. In opioid‐dependent patients, intraoperative low‐dose S‐ketamine significantly decreased postoperative morphine use, with long‐term benefits in back pain relief, ambulation, and disability scores [22]. Continuous epidural infusion of 0.1% ropivacaine provided superior pain relief and reduced systemic opioid needs with high patient satisfaction and comparable AEs [25]. Compared with remifentanil, dexmedetomidine in total intravenous anesthesia offered better pain control, reduced postoperative analgesic requirements, and lowered the incidence of postoperative nausea and vomiting [26]. In children undergoing spinal fusion, low‐dose intrathecal morphine (2 and 5 μg/kg) combined with PCA morphine improved analgesia, with 5 μg/kg also reducing intraoperative blood loss and no increase in side effects [28]. Overall, the individual RCTs within this most‐cited subset reported favorable analgesic and safety outcomes for multimodal approaches incorporating gabapentinoids, nonsteroidal anti‐inflammatory drugs (NSAIDs), *N*‐methyl‐*D*‐aspartate antagonists, *α*2‐agonists, local anesthetics, and intrathecal opioids in relation to postoperative pain control and opioid‐related AEs. These efficacy and safety statements originate from the included RCTs themselves and are summarized here to characterize the thematic content of the most influential literature; they are not conclusions of the present bibliometric analysis, which cannot appraise treatment effects.

**TABLE 2:** Top 10 studies.

Study	Title	Journal	Citations
Pandey et al. [20]	Evaluation of the optimal preemptive dose of gabapentin for postoperative pain relief after lumbar diskectomy: a randomized, double‐blind, placebo‐controlled study	Journal of Neurosurgical Anesthesiology	158
Hernández‐Palazón et al. [21]	Intravenous administration of propacetamol reduces morphine consumption after spinal fusion surgery	Anesthesia and Analgesia	125
Nielsen et al. [22]	Intraoperative ketamine reduces immediate postoperative opioid consumption after spinal fusion surgery in chronic pain patients with opioid dependency: a randomized, blinded trial	Pain	124
Pandey et al. [23]	Preemptive gabapentin decreases postoperative pain after lumbar discoidectomy	Canadian Journal of Anaesthesia/Journal Canadien D Anesthesie	94
Munro et al. [24]	Low‐dose ketorolac improves analgesia and reduces morphine requirements following posterior spinal fusion in adolescents	Canadian Journal of Anaesthesia/Journal Canadien D Anesthesie	90
Gottschalk et al. [25]	Quality of postoperative pain using an intraoperatively placed epidural catheter after major lumbar spinal surgery	Anesthesiology	85
Wonjung et al. [26]	Dexmedetomidine versus remifentanil in postoperative pain control after spinal surgery: a randomized controlled study	BMC Anesthesiology	83
Ozgencil et al. [27]	Perioperative administration of gabapentin 1200 mg day‐1 and pregabalin 300 mg day‐1 for pain following lumbar laminectomy and discectomy: a randomised, double‐blinded, placebo‐controlled study	Singapore Medical Journal	81
Gall et al. [28]	Analgesic effect of low‐dose intrathecal morphine after spinal fusion in children	Anesthesiology	80
Khurana et al. [29]	Postoperative pain and long‐term functional outcome after administration of gabapentin and pregabalin in patients undergoing spinal surgery	Spine	76

The dataset comprised 197 spinal surgery pain management studies. Overall, most studies enrolled adult participants, whereas adolescents and older adults were comparatively underrepresented. Participant sex and ASA classification were frequently incompletely reported, with nearly one‐quarter of studies omitting sex information and more than two‐thirds not reporting ASA status. Lumbar disc prolapse was the most frequently investigated pathology, followed by spondylolisthesis and spinal stenosis, while lumbar spinal fusion and lumbar discectomy were the predominant surgical procedures. Pain management interventions were mainly initiated during the postoperative or intraoperative period, and pharmacological approaches overwhelmingly predominated, whereas nonpharmacological interventions were only occasionally investigated (Table 3).

**TABLE 3:** Differences in characteristic distribution between Asian and non‐Asian groups.

Characteristics	Asia (*n* = 97)	Non‐Asia (*n* = 100)	*p*‐value
Publication, years	2016.53 ± 6.21	2011.30 ± 9.38	< 0.001
Sex, *n* (%)			0.205
Female	44 (45.36)	33 (33)	
Male	33 (34.02)	41 (41)	
NR	20 (20.62)	26 (26)	
Age, *n* (%)			< 0.001
Adolescent	2 (2.06)	13 (13)	
Adult	70 (72.16)	67 (67)	
Older	14 (14.43)	3 (3)	
NR	11 (11.34)	17 (17)	
Pain‐management timing, *n* (%)			0.318
Preoperative	14 (14.43)	11 (11)	
Intraoperative	32 (32.99)	26 (26)	
Postoperative	33 (34.02)	34 (34)	
Perioperative	18 (18.56)	29 (29)	
Intervention, *n* (%)			0.268
Anesthetics	35 (36.08)	28 (28)	
Opioids	26 (26.8)	32 (32)	
NSAIDs	16 (16.49)	19 (19)	
Antipyretic–analgesic drugs	4 (4.12)	10 (10)	
Corticosteroid	6 (6.19)	6 (6)	
SNRIs	2 (2.06)	2 (2)	
Nonopioid centrally acting analgesic	3 (3.09)	0 (0)	
Nondepolarizing neuromuscular blocker	1 (1.03)	0 (0)	
TENS	0 (0)	2 (2)	
Acupuncture	1 (1.03)	0 (0)	
Cold therapy	0 (0)	1 (1)	
Hypothermic therapy	1 (1.03)	0 (0)	
Music therapy	1 (1.03)	0 (0)	
Sacral canal therapy	1 (1.03)	0 (0)	

Abbreviations: NR = not reported, NSAIDs = nonsteroidal anti‐inflammatory drugs, SNRIs = serotonin–norepinephrine reuptake inhibitors, TENS = transcutaneous electrical nerve stimulation.

Pharmacological research clustered around five drug classes. Anesthetics were the most frequently investigated (63 studies), dominated by bupivacaine, ketamine, and ropivacaine, which together accounted for approximately three‐quarters of this class. Opioids followed (58 studies), with morphine alone representing more than half. NSAIDs (35 studies) centered on celecoxib, ketorolac, and parecoxib; antipyretic‐analgesic agents (14 studies) were almost exclusively paracetamol or acetaminophen; and corticosteroid trials (12 studies) predominantly used methylprednisolone or dexamethasone. Duloxetine accounted for all four serotonin–norepinephrine reuptake inhibitor (SNRI) studies, whereas nefopam, a nonopioid centrally acting analgesic, and rocuronium, a nondepolarizing neuromuscular blocker, were each evaluated in a single trial. Within every class, the remaining agents were investigated infrequently, indicating an evidence base that rests on a narrow set of well‐established drugs with only marginal exploration of alternative pharmacological pathways. Agent‐level frequencies are reported in Table 3.

Delivery routes, comparators, and outcome measures were similarly concentrated. Intravenous administration predominated (48.22%), followed by the oral (17.26%) and epidural (16.75%) routes, with every remaining route used in fewer than 8% of the studies. A placebo comparator was used in 58.38% of the trials and best supportive care in 1.52%. Pain assessment was strongly standardized around the VAS (77.16%), with the NRS a distant second (12.69%). Morphine consumption (34.52%), AEs following pharmacological intervention (34.01%), and postoperative supplementary analgesic consumption (22.34%) were the most frequently reported additional outcomes, whereas all other instruments, including spanning satisfaction, disability, physiological, sleep, and psychological domains, was applied in fewer than 10% of the studies. Outcome reporting beyond pain intensity was therefore heterogeneous and poorly standardized. Full distributions are presented in Table 3.

**FIGURE 4:**
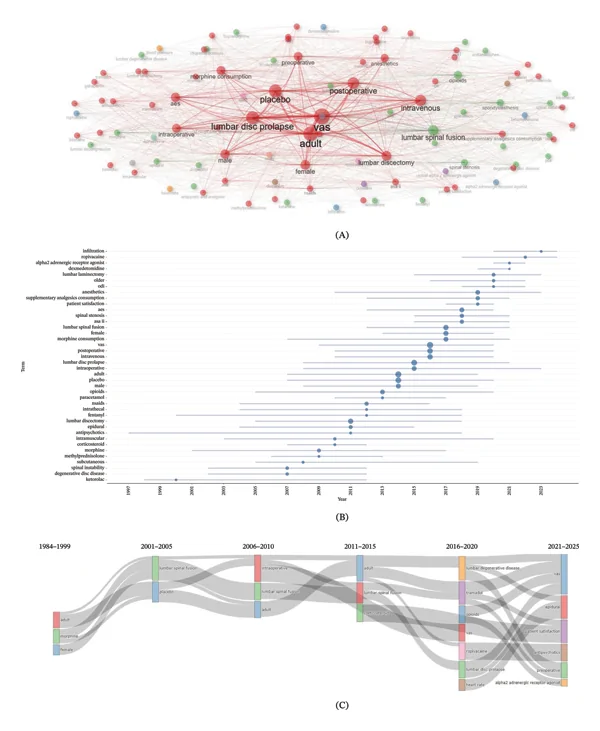
Keyword maps. (A) Keyword co‐occurrence map. Node size and line thickness indicate keyword and co‐occurrence frequencies, respectively. (B) Trend topics. Horizontal lines and nodes show the duration and median time of keyword appearances. (C) Thematic evolution. Each vertical bar represents keyword prevalence over time, with lines indicating the transition or continuity of terms across periods.

After removing the duplicates, 174 unique keywords were identified. Keyword co‐occurrence and thematic analyses highlighted the evolving research focus on pain management in spinal surgery (Figure 4). Before 2005, the focus was on evaluating the analgesic efficacy of ketorolac and its opioid‐sparing potential, as well as the use of antipsychotics for sedation and antiemetic purposes in adult spinal surgery patients [24, 30]. Between 2006 and 2010, lumbar discectomy and disc prolapse procedures were the focus, with placebo‐controlled trials assessing pain management using VAS [31–33]. In this period, perioperative analgesia through various routes was explored, including intravenous and epidural opioids, NSAIDs, and adjuncts like clonidine, considering the timing of drug administration (pre‐, intra‐, and postoperative) [31, 34]. From 2011–2015, research evolved toward more comprehensive approaches, emphasizing quantitative opioid‐sparing outcomes, such as morphine consumption and supplementary analgesic use, alongside the expansion of multimodal analgesia strategies, including corticosteroids and new administration routes like subcutaneous and intramuscular [21, 35, 36]. Between 2016 and 2020, the focus shifted to complex lumbar surgeries, particularly spinal fusion, and degenerative pathologies like spondylolisthesis and spinal stenosis [37–39]. Studies during this period expanded beyond pain scores, incorporating AEs and risk stratification, and more sophisticated multimodal analgesia regimens involving anesthetics, corticosteroids, NSAIDs, and antiepileptic agents were used [40–43]. Research was tailored to optimize analgesia for high‐complexity procedures through individualized risk assessment. Finally, from 2021–2025, the research pivoted toward patient‐centered trial designs tracking multidimensional outcomes such as pain intensity, opioid‐sparing metrics, functional recovery, and patient satisfaction [44–46]. High‐complexity spinal surgeries, especially lumbar spinal fusion, remain a central focus, with a refined emphasis on multimodal protocols and the safety of opioid use, including naloxone monitoring [47–49]. This period highlights the need for sex‐aware and risk‐stratified approaches to ensure optimal analgesia and recovery. Overall, the field progressed from foundational pain management and procedural comparisons to more personalized and multifaceted perioperative strategies, addressing the unique needs of patients undergoing complex spinal surgeries.

The analysis revealed significant differences between the Asian and non‐Asian groups in several aspects, including publication year and age (*p* < 0.001) (Table 3). Specifically, the mean publication year for the non‐Asian group (2011.30 ± 9.38) was significantly earlier than that of the Asian group (2016.53 ± 6.21). Regarding demographic characteristics, the Asian group had a slightly higher proportion of older individuals (14.43%), whereas adolescents (13.00%) were slightly higher in the non‐Asian group. However, the two groups did not significantly differ regarding sex, pain management timing, or intervention (*p* > 0.05). These findings indicate a pharmacology‐dominated evidence base, standardized VAS‐based assessment, and a temporal rather than conceptual regional divergence driven by a delayed Asian research surge.

## 4. Discussion

The clinical significance of lumbar postoperative pain lies in its impact on recovery, as persistent pain can reduce mobility, prolong disability, increase complication risk, delay rehabilitation, and diminish the quality of life, making effective pain management crucial for optimal postoperative outcomes. The pathological process of this pain involves muscle degeneration, fat infiltration, structural complications, central sensitization, and inflammatory responses, all contributing to persistent pain and reduced physical activity [50, 51]. Higher risk populations include patients with sarcopenia, osteoporosis, obesity, severe preoperative pain, disability, depression, or undergoing extensive surgery, and those with high multifidus muscle fat infiltration or pre‐existing central sensitization [9, 50, 51]. Multimodal analgesia is emphasized in treatment, combining nonopioid analgesics and preemptive regimens to optimize pain control and minimize opioid use [52]. NSAIDs, epidural analgesia, and local wound infiltration have been reported to be effective pharmacological interventions in previously published trials and syntheses [10, 53], while regional techniques like the erector spinae plane block and acupuncture [12, 54], along with nonpharmacological approaches, such as cognitive–behavioral‐based physical therapy, have been reported to be beneficial in managing postoperative pain in the primary literature [15, 55].

RCT publications for this condition have grown exponentially, driven by increasing research interest and advancements in surgery and pain management. However, citations per article declined from 2001–2012 because of higher publication volumes and a changing research focus. Five countries—the USA, Turkey, Iran, China, and Korea—contributed at least 10 lumbar postoperative pain RCTs, with the USA, Turkey, Iran, and Korea having the highest citations. The interpretations offered in the remainder of this paragraph are advanced as hypotheses rather than conclusions, because bibliometric indicators alone cannot test them. A plausible contributing factor for these countries may be their strong research infrastructure. However, China’s lower citation ranking has been hypothesized to relate to more limited international visibility, although this remains untested and could equally reflect differences in publication venue, database indexing coverage, or the more recent age structure of its research output [56–58]. The USA’s high citation rate may reflect its research leadership, and Spain’s peak citations are likely because of a few highly influential studies [23]. The predominance of authors contributing to only a single lumbar postoperative pain RCT is likely owing to the specialized nature of the research, multidisciplinary collaboration, and shifting research interests. One‐third of the RCTs on lumbar postoperative pain were published in leading journals owing to their high impact, rigorous review, and focus on relevant interdisciplinary topics.

The top 10 cited papers on lumbar postoperative pain RCTs focused on optimizing multimodal analgesia strategies intended to reduce pain, opioid consumption, and AEs. The findings reported within those trials concerned gabapentinoids, NSAIDs, S‐ketamine, epidural ropivacaine, and dexmedetomidine in relation to pain control and postoperative nausea [24, 25, 27]. The authors of those primary trials emphasized the safety and efficacy of combining various analgesics to improve postoperative outcomes while minimizing opioid‐related AEs; these are conclusions drawn within the individual RCTs and are not effectiveness estimates generated by the present bibliometric analysis [25, 27]. Adults were the primary focus of research on spinal surgery pain management, with a near‐equal representation of men and women, although some studies do not specify participant sex. Lumbar disc prolapse, spondylolisthesis, and spinal stenosis were mostly studied, with lumbar spinal fusion and lumbar discectomy being the most common surgical procedures [20, 21, 58]. However, older adults, adolescents, and patients with more severe ASA classifications were not sufficiently considered [22, 26]. Regarding pain management timing, most studies emphasize postoperative pain, with significant focus on the preoperative, intraoperative, and perioperative periods [20, 22, 58]. Beyond intervention type, underrepresentation of older adults, adolescents, and higher ASA patients persists despite their disproportionate perioperative risk and potential benefit from individualized opioid‐sparing strategies, limiting generalizability and highlighting a clear priority for future trial design.

The marked imbalance between pharmacological and nonpharmacological research warrants closer scrutiny, reflecting both prevailing clinical practice and structural biases within the evidence base. In the present corpus, nonpharmacological interventions were limited to a small number of trials, including transcutaneous electrical nerve stimulation and perioperative transcutaneous electrical acupoint stimulation [58, 59], both of which reduced postoperative opioid requirements, consensus‐based acupuncture associated with improved segmental analgesia [54], and cognitive–behavioral‐based physical therapy targeting pain catastrophizing and fear avoidance, which improved functional recovery in patients with chronic pain undergoing lumbar surgery [55]. Although these modalities align with the multifactorial structural, inflammatory, and psychosocial mechanisms of persistent postoperative pain and are associated with favorable safety profiles and minimal risk of dependence, the evidence remains sparse, methodologically heterogeneous, and limited by small sample sizes, short follow‐up, and limited blinding feasibility. This imbalance is unlikely to be incidental. It likely reflects structural drivers including ease of placebo‐controlled drug trial design, concentration of funding on pharmaceutical interventions, and publication and citation practices that preferentially amplify pharmacological research, which bibliometric indicators can reveal but cannot disentangle. Consequently, the observed dominance of pharmacological approaches may reflect publication and research emphasis rather than comparative clinical effectiveness. Given persistent postoperative pain despite technically successful surgery and the need to reduce opioid exposure, future trials should more rigorously evaluate psychological, physical, and neuromodulatory strategies within multimodal opioid‐sparing pathways using standardized outcome sets, adequate follow‐up, and formal risk‐of‐bias assessment.

Research on pharmacological interventions for lumbar postoperative pain primarily focused on anesthetics, with bupivacaine, ketamine, and ropivacaine being the most commonly studied [40, 57, 60]. Opioids, especially morphine, were also widely examined, along with other analgesics such as fentanyl and tramadol [61–63]. NSAIDs like celecoxib and ketorolac, and antipyretic–analgesic drugs, such as paracetamol and acetaminophen, were frequently investigated [35, 42, 64, 65]. Additionally, corticosteroids, primarily methylprednisolone and dexamethasone, were explored, as well as emerging treatments like SNRIs and nefopam and rocuronium, reflecting an expanding interest in alternative pharmacologic options for enhancing multimodal analgesia [36, 60, 64, 66]. Regarding drug administration routes, intravenous administration was the most common, followed by oral, epidural, and intramuscular routes. Placebo was the most frequently used control intervention, and pain levels were primarily assessed using the VAS, with other commonly measured outcomes such as morphine consumption, AEs, and postoperative analgesic use. Various additional outcome measures were employed, including patient satisfaction, disability indices, and various scales to assess pain, functional status, and psychological factors, indicating a comprehensive approach to evaluating the effectiveness of pain management strategies. Nonpharmacological interventions including acupuncture, transcutaneous electrical nerve stimulation, and cognitive–behavioral‐based physical therapy remain underrepresented compared with pharmacological strategies despite favorable safety and opioid‐sparing potential, indicating a need for greater research emphasis.

The evolution of lumbar postoperative pain research reflects the shifts in clinical priorities, responses to emerging issues, and advancements in surgery and pain management. Before 2005, the focus was on opioid‐sparing strategies, especially ketorolac, and antipsychotics, because of the lack of widespread recognition of the long‐term risks of opioids, leading researchers to explore alternatives for effective pain management and sedation [24, 30]. Between 2006 and 2010, as lumbar discectomy and disc prolapse surgeries became more common, the research focus was on perioperative analgesia, with a need to optimize pain management specific to these procedures and improve recovery [33, 59, 67]. The opioid crisis of the early 2010s spurred research from 2011 to 2015, prioritizing multimodal analgesia to reduce opioid use and exploring sex‐specific responses as part of the growing movement toward personalized medicine. Between 2016 and 2020, the rise in more complex spinal surgeries, including spinal fusion, necessitated more individualized pain management approaches that incorporated comprehensive risk assessment, multimodal regimens, and a broader range of outcomes to improve patient safety [37, 38, 62]. Finally, from 2020 to 2025, patient‐centered care emerged as the focus with a greater emphasis on improving functional recovery, patient satisfaction, and opioid safety, driven by the ongoing opioid epidemic and a growing understanding of the importance of personalized, holistic care to optimize recovery outcomes [45, 47, 49].

The regional and demographic associations presented below are based on descriptive bibliometric analyses and should therefore be interpreted as correlational rather than causal. The explanations proposed below are advanced explicitly as hypotheses: The present dataset contains no variables capable of testing them, and they should not be read as conclusions of this study. The earlier publication year for the non‐Asian group may reflect a longer history of lumbar postoperative pain research in Western countries with an established research infrastructure. The higher proportion of older individuals in the Asian group may plausibly relate to the aging populations of countries such as Japan and China, whereas the higher adolescent proportion in the non‐Asian group may stem from more focus on pediatric spinal conditions in Western countries. These differences may be shaped by regional healthcare priorities and research and development stages. In many Western countries, aging‐related conditions such as spinal disorders are hypothesized to have received earlier health‐policy attention, leading to earlier and more extensive research on lumbar postoperative pain and related surgeries. Meanwhile, many Asian countries, including Japan, may initially have focused more on other healthcare issues, such as infectious diseases, delaying the focus on spinal pain management. However, owing to the aging populations in these countries, a recent surge has emerged in research related to spinal health, although it is still catching up with the progress made in Western nations. This shift is evident from the growing emphasis on geriatric care and spinal surgery in China, South Korea, and India. These historical interpretations are consistent with the observed temporal pattern but were not directly examined here and would require bibliometric or health‐policy data specifically designed to test them.

Bibliometric analysis provides valuable insights into the trends and patterns of research on postoperative lumbar pain; nonetheless, some limitations should be considered. First, the reliability and completeness of the analysis are inherently dependent on the data sources and search strategy. Despite the use of a comprehensive retrieval approach, relevant studies may still have been missed due to differences in database coverage, indexing systems, search syntax, and keyword selection. In addition, variability in data preprocessing procedures, including record deduplication, author/keyword disambiguation, and terminology standardization, may have introduced a degree of bias, potentially affecting the completeness and consistency of the final dataset. Moreover, the restriction to English‐language RCTs may have further contributed to selection bias by excluding relevant studies published in other languages, particularly those from non–English‐speaking regions with substantial research output. Second, bibliometric methods are primarily based on publication counts and citation‐based indicators, which reflect research productivity and academic influence but do not allow for direct assessment of methodological quality, risk of bias, or clinical relevance of the included studies. Therefore, the observed publication and citation patterns should be interpreted with caution and not be considered proxies for evidence strength. Third, citation‐based metrics are subject to temporal bias, as earlier publications have had more time to accumulate citations, which may lead to an underestimation of the influence of more recent studies. Fourth, differences in publication practices across regions, such as language barriers and publication preferences, could also affect the representation of studies in certain countries, potentially skewing the findings. Furthermore, the evolving nature of research methodologies or heterogeneity of study designs was not considered, possibly influencing the interpretation of trends over time. Finally, global research patterns were examined, although they may not have fully addressed the underlying reasons for these regional differences, such as cultural, economic, and healthcare system variations that shape research priorities. Accordingly, the regional and historical explanations offered in the Discussion should be regarded as hypotheses generated by, rather than conclusions derived from, the present bibliometric data.

## 5. Conclusion

RCTs on lumbar postoperative pain have grown significantly, with a decline in citations per article because of a higher publication volume. Leading countries, such as the USA, Turkey, Iran, and Korea, dominate the research, whereas China ranked lower on citation‐based indicators; limited international visibility is a plausible hypothesis for this pattern that could not be tested with the present data. One‐third of the studies were published in high‐impact journals, reflecting the specialized and interdisciplinary nature of the research. Research in this field has evolved from opioid‐sparing strategies to multimodal, personalized, and patient‐centered care, driven by the opioid crisis and surgical advancements, with research timelines and demographics possibly reflecting regional healthcare priorities, where Western countries have focused earlier on aging‐related spinal conditions and Asian countries are now catching up, an interpretation that should be regarded as a hypothesis for further study. Future research should advance multimodal personalized pain management, address regional disparities, and focus on aging populations to reduce opioid use and improve patient outcomes.

## Author Contributions

Duoduo Li and Yena Gan contributed to the study concept and design. Changxin Liu and Xing Yu supervised the study. Xia Li, Caoyuan Yang, Yujia Zhang, Ranya Huang, Qifei Li, Anagul Tudi, and Bofei Meng collected the data. Duoduo Li and Yena Gan analyzed the data. Duoduo Li, Yena Gan, and Xia Li contributed to the data interpretation. Duoduo Li and Yena Gan drafted the manuscript. Sheng Han and He Zhu reviewed the manuscript.

## Funding

This research did not receive any specific grant from funding agencies in the public, commercial, or not‐for‐profit sectors.

## Disclosure

All the authors approved the final version of the manuscript.

## Ethics Statement

The authors have nothing to report.

## Consent

The authors have nothing to report.

## Conflicts of Interest

The authors declare no conflicts of interest.

## Supporting Information

Additional supporting information can be found online in the Supporting Information section.

## Supporting information


**Supporting Information** Additional file 1. Table 1. The search strategy of pain management after lumbar spine surgery.

## Data Availability

All data generated or analyzed during this study are included in this published article and supporting file.
