# Supplementary material for: Application of Pain Management After Lumbar Spine Surgery: A Bibliometric Analysis of Randomized Controlled Trials
Source: Pain Res Manag. 2026 Sep 8;2026:3601838. doi: 10.1155/prm/3601838 (PMC13551594; doi:10.1155/prm/3601838)
Supplement: Supplementary file 1 [file PRM-2026-3601838-s001.docx]

**TABLE 1.** The search strategy of pain management after lumbar spine surgery

| **Database** | **Retrieval formula** | **Results (2025-05-01)** |
| --- | --- | --- |
| Scopus | #1 TITLE-ABS-KEY = (dorsalgia or backache or coccyx or coccydynia or sciatica or spondylosis or ‘lumbar pain’ or lumbago or discitis or (disc and (degeneration or prolapse or herniation)) or ‘facet joints’ or arachnoiditis or (spinal and (fusion or neoplasms)) or ‘intervertebral disk’)  #2 TITLE-ABS-KEY = (postoperative or ‘post operative’ or post-operative)  #3 TITLE-ABS-KEY = (randomized or randomised or placebo or randomly or trial or groups)  #4 #1 and #2 and #3 | 933 |
| Web of Science Core Collection (WoSCC) | #1 TS = (dorsalgia or backache or coccyx or coccydynia or sciatica or spondylosis or (lumbar adj pain) or lumbago or discitis or (disc adj (degeneration or prolapse or herniation)) or (facet adj joints) or arachnoiditis or (spinal and (fusion or neoplasms)) or ‘intervertebral disk’)  #2 TS = (postoperative or ‘post operative’ or post-operative)  #3 TS = (randomized or randomised or placebo or randomly or trial or groups)  #4 #1 and #2 and #3 | 7,503 |
| PubMed (MEDLINE) | #1 "dorsalgia"[Title/Abstract] OR "backache"[Title/Abstract] OR "coccyx"[Title/Abstract] OR "coccydynia"[Title/Abstract] OR "sciatica"[Title/Abstract] OR "spondylosis"[Title/Abstract] OR ("lumbar"[Title/Abstract] adj "pain"[Title/Abstract]) OR "lumbago"[Title/Abstract] OR "discitis"[Title/Abstract] OR ("disc"[Title/Abstract] adj ("degeneration"[Title/Abstract] OR "prolapse"[Title/Abstract] OR "herniation"[Title/Abstract])) OR ("facet"[Title/Abstract] adj "joints"[Title/Abstract]) OR "arachnoiditis"[Title/Abstract] OR ("spinal"[Title/Abstract] AND ("fusion"[Title/Abstract] OR "neoplasms"[Title/Abstract])) OR "intervertebral disk"[Title/Abstract]  #2 "postoperative"[Title/Abstract] OR "post-operative"[Title/Abstract] OR "post-operative"[Title/Abstract]  #3 "randomized"[Title/Abstract] OR "randomised"[Title/Abstract] OR "placebo"[Title/Abstract] OR "randomly"[Title/Abstract] OR "trial"[Title/Abstract] OR "groups"[Title/Abstract]  #4 #1 and #2 and #3 | 3,009 |
| CENTRAL (The Cochrane Library) | #1 (dorsalgia or backache or coccyx or coccydynia or sciatica or spondylosis or (lumbar adj pain) or lumbago or discitis or (disc adj (degeneration or prolapse or herniation)) or (facet adj joints) or arachnoiditis or (spinal and (fusion or neoplasms)) or ‘intervertebral disk’):ti,ab,kw  #2 (postoperative or ‘post operative’ or post-operative):ti,ab,kw  #3 (randomized or randomised or placebo or randomly or trial or groups):ti,ab,kw  #4 #1 and #2 and #3 | 2,741 |
| Embase | #1 dorsalgia:ti,ab,kw OR backache:ti,ab,kw OR coccyx:ti,ab,kw OR coccydynia:ti,ab,kw OR sciatica:ti,ab,kw OR spondylosis:ti,ab,kw OR (lumbar:ti,ab,kw adj pain:ti,ab,kw) OR lumbago:ti,ab,kw OR discitis:ti,ab,kw OR (disc:ti,ab,kw adj (degeneration:ti,ab,kw OR prolapse:ti,ab,kw OR herniation:ti,ab,kw)) OR (facet:ti,ab,kw adj joints:ti,ab,kw) OR arachnoiditis:ti,ab,kw OR (spinal:ti,ab,kw AND (fusion:ti,ab,kw OR neoplasms:ti,ab,kw)) OR ‘intervertebral disk’:ti,ab,kw  #2 postoperative:ti,ab,kw OR ‘post operative’:ti,ab,kw OR post-operative:ti,ab,kw  #3 randomized:ti,ab,kw OR randomised:ti,ab,kw OR placebo:ti,ab,kw OR randomly:ti,ab,kw OR trial:ti,ab,kw OR groups:ti,ab,kw  #4 #1 and #2 and #3 | 4,342 |
